# The Research Committee's Future

**Published:** 1919-03-22

**Authors:** 


### FREEDOM IN MEDICAL INVESTIGATION.

### The Research Committee's Future.

In the course of the debate on the second reading of the Ministry of Health Bill on February 26 suggestions were made that it would be preferable to place the Medical Research Committee under the direct control of the Minister of Health rather than, as proposed in the Bill, to reconstitute it under the direction of a committee of the Privy Council on the principle already adopted with the Advisory Council for Scientific and Industrial Research. In a recently published memorandum, with an appendix by Sir W. M. Fletcher, K.B.E., F.R.S., Secretary to the Medical Research Committee, Dr. Addison states with admirable clearness his reasons for the original proposal.

The problem lies in the question of the exact extent of research which should preferably be carried out by a body in close relation to the executive side of the Ministry, and how much by a staff whose efforts are not necessarily directed by health matters under immediate administration. The object must be to prevent overlapping, limitation of activities, and restriction of free communication with all similar scientific bodies the world over. In the past the Research Committee has been under the control of the Minister responsible for Health Insurance, so that he could defend their interests in Parliament, but in practice this official has relied upon the Committee's own selection of its fields of work, expenditure schemes, etc., and the acknowledgments published in the Committee's first annual report suggest the satisfactory nature of this arrangement. Why, then, it may be asked, should reorganisation be necessary and an exactly parallel policy be impossible to follow? According to present plans there will be established a Ministry for England and Wales only, with resulting readjustments as regards Health Insurance in Scotland and Ireland, but the Medical Research Fund has from the first been made single for the United Kingdom. If the Committee's position remains unchanged the administrative and scientific interests would in future undoubtedly clash, since, for the latter, it is absolutely essential that the closest touch be maintained with the best scientific activities, even other than medical, in all parts of the kingdom, and that no executive barriers be imposed.

A progressive Ministry must inevitably become, from time to time, deeply committed to particular systems of health administration. It is essential that the work of the Committee should be free and uncontrolled by influence arising in such a way, and to secure this, relations with the Ministry must be made as elastic as possible. A direct and special attachment would tend completely to absorb scientific activities upon problems which appeared for the moment to be politically urgent, and although inquiries started with a purely practical object have frequently led medical men into discoveries quite distinct from the original problem, yet the needed solution has frequently arisen quite unexpectedly from other researches conducted on the promptings of science alone.

Another argument against the close association of the Committee with any single strong administrative body lies in the risk of loss in confidence from the many other departments which have in the past availed themselves of their investigations and advice. Such procedure would at least limit the access of the Committee to confidential proceedings of many bodies whose sympathetic co-operation is essential to any national research organisation, the keynote of which must be the free and closest association of all branches of scientific work.

Dr. Addison proposes that the best relationship to adopt will be representation to a Committee of the Privy Council under the Lord President, who would be the Minister responsible to Parliament for the expenditure of the moneys involved, and if he remain in future a member of the House of Lords, one of the Ministerial members of the Privy Council Committee could be made answerable for medical-research matters in the House of Commons. To such a course there are several outstanding advantages. The Privy Council is the only Department which has an Imperial range, and already analogous research bodies are organised by the Canadian, South African, and Australian Governments. The Medical Research Committee would remain in a position to serve all administrative sections alike without undue influence arising from or upon any one of them, and were all research bodies, other than purely medical, associated under a similar type of Government control, interchange and assimilation of investigational work among them would lead to greatly increased efficiency and progress.

In his appendix Sir W. M. Fletcher gives some convincing examples of the manner in which what may be called the by-products of research have been turned to world-wide use. In fact, their reading suggests, in some instances, that the unexpected result has proved of far greater value than the solution of the original problem for which the work was undertaken, but the real lesson to be derived concerns the enormous advantage which lies in the perfected system of complete availability to other men of details of any experimental work, no matter upon what object directed.

The need is for centralisation, accessibility of results, and, above all, freedom of action. Expenditure and financial considerations are wisely left to Government control and representation, but the scientific mind must, like the artistic, be unfettered if it is to give its best results. Should all these proposals for the coming Ministry be put forward with their outlined purpose genuinely in view, the scheme appears to deserve general commendation, and the Medical Research Committee should continue their career of admirable and unrestricted accomplishment.

